# Network Approach to Understanding Emotion Dynamics in Relation to Childhood Trauma and Genetic Liability to Psychopathology: Replication of a Prospective Experience Sampling Analysis

**DOI:** 10.3389/fpsyg.2017.01908

**Published:** 2017-11-02

**Authors:** Laila Hasmi, Marjan Drukker, Sinan Guloksuz, Claudia Menne-Lothmann, Jeroen Decoster, Ruud van Winkel, Dina Collip, Philippe Delespaul, Marc De Hert, Catherine Derom, Evert Thiery, Nele Jacobs, Bart P. F. Rutten, Marieke Wichers, Jim van Os

**Affiliations:** ^1^Department of Psychiatry and Psychology, Maastricht University Medical Centre, Maastricht, Netherlands; ^2^Department of Psychiatry, Yale School of Medicine, New Haven, CT, United States; ^3^University Psychiatric Centre KU Leuven, Leuven, Belgium; ^4^Centre of Human Genetics, University Hospitals Leuven, KU Leuven, Leuven, Belgium; ^5^Department of Obstetrics and Gynecology, Ghent University Hospitals, Ghent University, Ghent, Belgium; ^6^Department of Neurology, Ghent University Hospital, Ghent University, Ghent, Belgium; ^7^Faculty of Psychology and Educational Sciences, Open University of the Netherlands, Heerlen, Netherlands; ^8^Department of Psychiatry, Interdisciplinary Center Psychopathology and Emotion Regulation, University of Groningen, University Medical Center Groningen, Groningen, Netherlands; ^9^Department of Psychosis Studies, Institute of Psychiatry, King's Health Partners, King's College London, London, United Kingdom; ^10^Department of Psychiatry, Brain Centre Rudolf Magnus, University Medical Centre Utrecht, Utrecht, Netherlands

**Keywords:** emotion dynamics, directed, weighted, network, time-series, genetic, psychopathology, childhood trauma

## Abstract

**Background:** The network analysis of intensive time series data collected using the Experience Sampling Method (ESM) may provide vital information in gaining insight into the link between emotion regulation and vulnerability to psychopathology. The aim of this study was to apply the network approach to investigate whether genetic liability (GL) to psychopathology and childhood trauma (CT) are associated with the network structure of the emotions “cheerful,” “insecure,” “relaxed,” “anxious,” “irritated,” and “down”—collected using the ESM method.

**Methods:** Using data from a population-based sample of twin pairs and siblings (704 individuals), we examined whether momentary emotion network structures differed across strata of CT and GL. GL was determined empirically using the level of psychopathology in monozygotic and dizygotic co-twins. Network models were generated using multilevel time-lagged regression analysis and were compared across three strata (low, medium, and high) of CT and GL, respectively. Permutations were utilized to calculate p values and compare regressions coefficients, density, and centrality indices. Regression coefficients were presented as connections, while variables represented the nodes in the network.

**Results:** In comparison to the low GL stratum, the high GL stratum had significantly denser overall (*p* = 0.018) and negative affect network density (*p* < 0.001). The medium GL stratum also showed a directionally similar (in-between high and low GL strata) but statistically inconclusive association with network density. In contrast to GL, the results of the CT analysis were less conclusive, with increased positive affect density (*p* = 0.021) and overall density (*p* = 0.042) in the high CT stratum compared to the medium CT stratum but not to the low CT stratum. The individual node comparisons across strata of GL and CT yielded only very few significant results, after adjusting for multiple testing.

**Conclusions:** The present findings demonstrate that the network approach may have some value in understanding the relation between established risk factors for mental disorders (particularly GL) and the dynamic interplay between emotions. The present finding partially replicates an earlier analysis, suggesting it may be instructive to model negative emotional dynamics as a function of genetic influence.

## Introduction

There is a growing interest in understanding the role of daily-life emotion dynamics underlying psychopathology (van Os et al., 2017). Emotions are considered promising candidates for the study of mechanisms underlying the early expression of subthreshold mental phenomena. From a complex dynamic system theory perspective, alterations in personal emotion dynamics may serve as an early warning sign for a tipping point signaling a transition from a subthreshold state to a clinical state—akin to an electrical signal in epilepsy that is monitored to detect the tipping point before a convulsion (Wichers et al., 2015; Nelson et al., 2017).

In this regard, the network approach provides a useful analytical strategy to gain insight into modeling interactive emotion dynamics, and identifying highly connected emotions that are critical in predicting transition to a more severe state. In recent years, the network approach to psychopathology has brought a novel perspective to conceptualizing mental disorders. Network studies investigate the network of symptoms mutually impacting each other in a variety of mental disorders such as depression and psychotic disorder (Borsboom, 2017). However, one of the primary challenges for the network investigation is that most studies rely on static observations (signs and symptoms) collected from samples with static states (mental disorders) to master a highly fluid phenomenon (Guloksuz et al., 2017).

The experience sampling method (ESM) is designed to prevent recall bias by capturing emotions in real time. ESM uses a rigorous structured diary method for intensive collection of emotions (e.g., sadness, cheerfulness) at random moments during the day, during a certain period (days or weeks), thus providing the essential platform for gathering data for emotion dynamics research (Verhagen et al., 2016).

Recently, the field has advanced to network analysis of ESM data (Pe et al., 2015; Bringmann et al., 2016; Klippel et al., 2017). Emotions have been found to interact with each other in the network, in which momentarily assessed emotions are represented by a node and the predictive regressive association of that emotion at moment *t–*1 on the same or another emotion at the subsequent moment *t*, is represented by an edge (Borsboom and Cramer, 2013; Schmittmann et al., 2013). Previous studies demonstrated that an increase in connectivity between affective states was associated with an increased risk for mental disorders (Wichers et al., 2016). Utilizing this approach, the persistence of an emotion over time—inertia—was found to be associated with both current and future depressive episodes (Wichers et al., 2016). By analyzing the auto-regressive coefficient of the emotion, inertia can be studied applying the time series network approach (Kuppens et al., 2012; Bringmann, 2016; Bringmann et al., 2016).

There is growing evidence that the impact of environmental exposure spreads through the symptom network and increase the level of admixture rather than impacting on a symptom domain (Smeets et al., 2012; van Nierop et al., 2014; Guloksuz et al., 2015, 2016). Using data from the general population, previous network investigations showed that the associations between symptoms dimensions and network density increased as a function of the level of environmental exposure (Isvoranu et al., 2016). In a similar fashion, there is some evidence that familial vulnerability operates on increasing connections between symptoms, which in turn leads to a more static and persistent clinical state (Smeets et al., 2014). Given these findings, we previously investigated the network structure of emotional dynamics across environmental and genetic vulnerability strata in a female-female twin population (Hasmi et al., submitted). Although, some differences were observed in the network structure between groups that might be suggestive of an increase in connectivity as a function of vulnerability, findings in general were inconclusive. We now have collected a second large twin sample which can serve as a replication of the previous study in analyzing the impact of vulnerability on emotion dynamics (Hasmi et al., submitted). The present study therefore investigated in a general population mixed-gender twin sample whether genetic liability to psychopathology and childhood trauma (hereafter referred to as “GL” and “CT”, respectively) are associated with the network structure of individual emotions—“cheerful,” “insecure,” “relaxed,” “anxious,” “irritated,” and “down”—collected using the ESM method.

In summary, our contributions in the present article are as follows:

- The current study is the first to attempt to replicate a previous network study investigating the question whether genetic factors and exposure to childhood trauma are associated with an alteration in inner psychological functioning at the level of emotions in daily life using the ESM method. Our focus on the emotional dynamics is based on evidence that these dimensions are sensitive to stress exposure and are altered in numerous stress-related mental disorders. It is hypothesized that the higher the levels of risk, the more the emotional network is connected, particularly as regards the negative emotions.
- We utilize a statistical method that is suited to extract and compare network structural parameters from ESM data at the population level dealing with computer tractability matters due to big samples with complex regression models. We inferred network models from the data by assimilating the predictive value of one variable on the other in the time after, to the average connection strengths between our variables of interest.
- We attempt to explain how the impact of genes and early environmental exposure on emotional networks could further be illustrated by examining the moderating effects of genetic risk for psychopathology and childhood trauma on the connections in the network and on three network structural indices. To better evaluate the validity of this approach, we replicate the method in this paper for visualizing the effect of genes and trauma on the network first by displaying visually three different emotional networks of subgroups of participants with increasing childhood trauma and in three emotional networks of subgroups with increasing genetic risk for psychopathology, and second by statistically comparing all those network parameters between the subgroup's networks using permutation testing.

## Methods

### Participants

The study sample was derived from the East Flanders Prospective Twin Study register, a population-based prospective register, recording all multiple births in Flanders, Belgium, since 1964 (Derom et al., 2013). Zygosity was determined through sequential analysis based on sex, fetal membranes, umbilical cord blood groups, placental alkaline phosphatase, and DNA fingerprints. Individuals who were registered in the EFPTS and who fulfilled the inclusion criteria were invited to participate in the TwinssCan project, a longitudinal study collecting data on adolescents and young adults between the ages of 15 and 35 years, including twins, their siblings, and parents. The TwinssCan project, which started enrollment in April 2010, is a general population based, ongoing longitudinal study (Derom et al., 2013; Pries et al., 2017). Participants were included if they understood the study procedure and were able to provide valid, reliable, and complete data. All participants gave written informed consent. For participants below the age of 18 years, parent(s) also signed an informed consent form. Participants were excluded if they had a pervasive mental disorder as indicated by caregivers. The local ethics committee (Commissie Medische Ethiek van de Universitaire ziekenhuizen KU Leuven, Nr. B32220107766) approved the study. For the present study, only twins and siblings who completed the ESM protocol were analyzed, leaving 740 participants.

### Measurements

#### Experience sampling method (ESM)

Before the start of the study, the ESM procedure was explained to the participants during an initial briefing session, and a practice trial was performed to confirm that participants were able to understand the 7-point Likert scale response format. During these sessions, subjects were also instructed to complete their reports immediately after the beep, thus minimizing memory distortion. At the start of the protocol, participants received a PsyMate, a custom-made electronic medical Personal Digital Assistant with a touch screen, which was designed to emit a beep-signal at random moments within each of ten 90-min intervals between 07.30 a.m. and 10.30 p.m. on 6 consecutive days. The semi-random beep design prevents participants from anticipatory behaviors and has proven superiority in self-reported adherence in a previous study (Jacobs et al., 2005; Verhagen et al., 2016). At each beep-signal, participants were asked to stop their activity and to enter their current thoughts, context (activity, persons present, and location), appraisals of current situation and mood. To assure reliability and validity, as described in detail before (deVries and Delespaul, 1989; Jacobs et al., 2005), the Psymate records the time at which participants completed the assessment. Furthermore, each beep-signal was accompanied by a 15-min window in which the questionnaire was available to the participant. Reports were required to be completed within 15 min of the beep, with the data recorded as missing outside that interval, as previous work has shown that outside this interval, reports are less reliable and, therefore, less valid (Delespaul, 1995). Also, subjects with fewer than 20 reports were excluded from the analysis.

The items collected by ESM consist of around 40 variables indexing thoughts, current context (activity, social context, and location), appraisals of the current situation, and Emotions. Emotion items at each beep were rated by participants on 7-point Likert scales ranging from 1 = “not at all” to 7 = “very.” As in the original study, only 6 emotion variables were chosen for analysis, given their maximum within-person time-lagged variability and therefore minimal floor effect, and given their covering of the whole emotional and core affect spectrum (Russell, 2003). This resulted in the selection of the following emotion items: “cheerful” (positive valence, high arousal), “relaxed” (positive valence, low arousal), “irritated” (loading in both the negative and the positive affect dimensions, high arousal), “down” (negative valence, low arousal), “insecure” and “anxious” (negative valence, high arousal; Hasmi et al., submitted).

#### Childhood trauma

The variable CT was assessed using the shortened 25 item version of the 70-item Childhood Trauma Questionnaire (Bernstein et al., 1994, 2003). The CTQ-SF is widely used and validated in various languages including Dutch (Bernstein et al., 2003; Thombs et al., 2009). The continuous variable “CT” reflected the total score of the 25 items on the questionnaire. To visualize the effect of CT on the network, the CT variable was recoded into three categories indexing increasing levels of CT total score and, therefore, severity of trauma (tertile groups). The regression coefficients, for the predictive association between the lag and the current emotions, were calculated for each of the three CT strata before being represented graphically as a network and compared (see below).

#### SCL-90-R

The Symptom Checklist-90-R (SCL-90-R), a reliable and valid self-report instrument for screening a range of symptoms occurring in the past week, was used to index the overall severity of psychopathology (Wigman et al., 2013). The SCL-90-R consists of nine subscales (Somatization, Obsessive-compulsive, Interpersonal-sensitivity, Depression, Anxiety, Hostility, Phobic anxiety, Paranoid Ideation, and Psychoticism), covering the entire range of psychopathology. The SCL-90-R was assessed twice within an interval of 6 months. First, scores were averaged per participant. Consistent with previous analyses (Wigman et al., 2013), a dichotomous measure of SCL-90-R was used in the analyses, based on the arbitrary cut-off point of 75th percentile. The resulting two-level variable (“SCL-severity”) reflected the levels of severity of psychopathology (Wigman et al., 2013).

#### Genetic liability to psychopathology

Genetic liability to psychopathology was determined on the basis of the SCL-90 value (i.e., “low” or “high” psychopathology) in the co-twin and zygosity status, consistent with previous work (Kendler et al., 1995; Wichers et al., 2007; Kramer et al., 2012). This procedure resulted in three categories of “genetic liability”: participants with co-twins having a low level of psychopathology (the reference category at lowest genetic liability); participants with a dizygotic (DZ) co-twin with a high level of psychopathology (intermediate level of genetic liability for psychopathology) and participants having a monozygotic (MZ) co-twin with a high level of psychopathology (highest level of genetic liability for psychopathology).

### Statistical analysis

All analyses were performed using Stata version 14.0. (StataCorp, College Station, TX, USA). To take into account the hierarchical structure of the data, multilevel (mixed-effects) linear regression models were fitted using the XTMIXED procedure in Stata, considering that level-one units (multiple observations per individual) clustered into level-two units (level of individual twins), that were nested within level-three units (twin pairs).

### Associations between *t*-1 emotional states and current emotional states

Time-lagged variables were used as predictors in the multilevel models (Bringmann et al., 2013). Cheerful at time *t* was predicted by (i) “cheerful,” (ii) “relaxed,” (iii) “irri*t*ated,” (iv) “insecure,” (v) “anxious,” and (vi) “down” at *t*−1 (lag 1). All lagged variables were person mean-centered to disentangle within-subject from between-subject effects, which is now the standard procedure in the field of network analyses (Wang and Maxwell, 2015). The same analysis was performed for each of the other emotional states at time point t (dependent variable) in six separate models. Thus, the six affective states variables at *t* were predicted by all six emotion variables at *t*−1. All lagged emotion variables were entered simultaneously in the model, to assess their independent effects. One example of a regression model is:

Cheerful_ijk_ = (B0+e_ijk_) + B1 ^*^ lag cheerful_ijk_ + B2 ^*^ lag insecure_ijk_ + B3 ^*^ lag relaxed_ijk_ + B4 ^*^ lag anxious_ijk_ + B5 ^*^ lag irritated_ijk_ + B6 ^*^ lag down_ijk_ + (B7+u_7ijk_) ^*^ time_ijk;_

Where B0 is the intercept, B1–B7 stand for the regression coefficients, the subscript *i* stands for the assessment level, *j* for individuals, *k* for twin pairs and u7_ijk_ for the random slope of time (see next sub section), and time is the beep number over days (1–50).

As the time between lagged and current moment must be contiguous, and all beep moments were in the waking time period of the day, the first beep of the day was excluded in all analyses. Analyses were performed across 3 strata of CT as well as across 3 strata of genetic vulnerability.

### Random slope of time

A time variable (i.e., beep number, counting from 1 to 50) was included in all regression models since any association in the network can be interpreted only if no systematic trend is present in the data (i.e., the models are controlled for time effects). Because any trend that may be present could differ across participants, a random slope for time was added to the models at the individual level, representing the standard procedure for analysis in network research (Wang and Maxwell, 2015).

### The construction of emotion networks

A complete set of analyses in one stratum yielded 36 unstandardized regression coefficients (B). These coefficients were represented in a graph using the following procedure:

A 6-by-6 matrix with the regression coefficients (B) was constructed. The connection thus denotes the extent to which the emotion variable (e.g., cheerful) at time point *t*−1 predicts another emotion item (e.g., relaxed; ➔*B*_*cheerful*−*relaxed*_) at time point *t*, while controlling for all other variables. The elements on the diagonal are the autoregressive effects (self-loops, e.g., *B*_*cheerful*−*cheerful*_).

This procedure was applied across the 3 strata of CT and the 3 strata of GL, separately (in total 6 graphs). Visualization of networks was obtained using R (qgraph package; Epskamp et al., 2012). Moreover, a value higher than the maximum absolute value of the whole set of regression coefficients, in the 3 strata of CT and then in the 3 strata of GL, was assigned to the argument “maximum” in qgraph to scale the connections widths to allow for a visual comparison across each set of 3 networks (Epskamp et al., 2012).

### Assessment of the network structure: density and node centrality

In addition to the individual connections in the network, overall measures can contribute to insight into the differences between networks. Density -also called overall connectivity- is the average of the absolute values of all regression coefficients in each of the networks. Following previous literature that examined the vulnerability underlying emotion density specifically at the level of personality dimensions (neuroticism), using time series networks, two parameters were calculated. Negative density is the average of regression coefficient absolute values, that have both the outcome and the predictor as a negative emotion (“anxious,” “irritated,” “insecure,” “down”). Positive density is the average of regression coefficient absolute values, that have both the outcome and the predictor as a positive emotion (“cheerful,” “relaxed”; Bringmann et al., 2016).

Centrality analyses allow for the identification of nodes- emotion items- that are more “central” than others in the network. According to the network theory of psychopathology, the greater the value of a node centrality index, the greater the probability for that node to activate other nodes in the network and create a “domino effect” that would activate a sequence of emotions, negative or positive depending on the connections of that node, from where it is difficult for individuals to get out from (Borsboom and Cramer, 2013). Two well-known centrality indices were calculated per network, allowing for a descriptive comparison across the three genetic liability and the three trauma strata: inward strength and outward strength centrality (Opsahl et al., 2010; Epskamp et al., 2012). In-strength of a certain node is the sum of all edges' weights toward it (that node is the outcome variable). The out-strength of a particular node is the sum of all edges' weights going from it (that node is the independent variable) The first will inform on which affect is the more regulated in the daily emotional experience, and the second on which is the more impactful among the six emotions in the daily life experience. Self-loops (e.g., regression weight between e.g., down at *t*−1 and down at *t*) are counted both in the inward and in the outward strength, taking into account the fact that self-loops are good indicators of emotion inertia, previously described as an indicator of increased vulnerability and decreased psychological flexibility (Hollenstein, 2015; Wichers et al., 2015).

All density and centrality parameters were calculated using Stata 14.0 (StataCorp, College Station, TX, USA). Permutation testing was used to calculate *p*-values for comparing them across strata (see below).

### Permutation testing

Mixed-effects models should ideally include random slopes for all time-varying predictor variables (and use fully unstructured covariance matrices for the random effects; Barr et al., 2013). This procedure allows for standard errors, and thus *p*-values, to be correctly estimated. However, the approach is not feasible in the present context, due to the large number of parameters needed, given that the covariance is unstructured (attempts to fit such models result in convergence problems). Therefore, a single random slope for *time* was included in the model (see above) and in order to obtain valid *p*-values, the statistical significance of regression coefficients was examined using permutation tests.

Two different types of permutation tests were performed. The first type was used to obtain valid *p*-values for each regression coefficient (edge). The second type was performed to compare regression coefficients across different strata of GL and CT.

For the first set of permutations, the value of the outcome variable (e.g., “cheerful” at *t*) was removed from each record of the original data file and reassigned to the same participant in random order in a copy of the original data set. Because assessments were shuffled within participants, the level of clustering within the data described above was unchanged. Refitting the model based on the permuted data then provides estimates of the model coefficients under the null hypothesis of no association. By repeating this process more than 1,000 times, a distribution of the regression coefficients under the null hypothesis was generated. Then, the observed coefficients were compared with the respective null hypothesis distributions to obtain *p*-values (i.e., the proportion of times that the coefficient in the permuted data was as large as or larger than the observed coefficient; multiplied by two to obtain a two-sided *p-*value). Given 2 × 3 × 6 × 6 tests for statistical significance, Simes correction for multiple testing was applied (Simes, 1986). Graphs derived from the analyses are shown both before and after Simes correction for multiple testing (alpha = 0.0224). While main results are the Simes corrected slopes, presentation of the figures with all the slopes prevents conclusions being directly drawn on differences that are merely the result of differences in power related to sample size in subgroups during the calculation of the *p*-values.

In the second set of permutations, the values of the CT variable were randomly assigned to the participants in another copy of the original data set. Again, regression coefficients in the original data were compared with regression coefficients under the null hypothesis of no difference in regression coefficients between the CT strata. With this procedure, all regression coefficients of the 36 connections (edges) in the network were tested for differences between the CT strata, regardless of the level of significance obtained with the first type of permutation testing. The same procedure was repeated for the different strata of genetic liability. Again, Simes correction for multiple testing was applied for individual edge differences (alpha 0.000462).

The same permutation testing procedure was applied in order to compare density as well as inward and outward strength parameters between the strata. Assuming independence between each index calculation, no multiple testing correction was applied.

## Results

### Sample characteristics

GL analyses included 598 participants (230 monozygotic and 368 dizygotic), given that participants without information on their zygosity status, non-twin siblings, and participants without information on psychopathology in the co-twin were excluded. CT analyses were performed with 688 individuals. Mean age of the participants was 17.6 years (*SD* 3.7). Forty percent of the total sample was male. The majority was still living with their parents (86%) and went to school (90%). In addition, 28% had a bachelor degree while only 5% had a low level of education.

The average CTQ-SF sum score was 33.8 (*SD* 8.1). Demographic data and mean levels of ESM items per subgroup of CT and GL are presented in Table 1. In general, the mean level of emotions in the third CT strata was significantly higher than in the first and the second strata. “Down” also differed between the second and the first strata. Except for the difference in “relaxed” between the third and the first strata, there were no differences between the GL strata.

**Table 1:** Descriptives stratified by childhood trauma and genetic liability.

**CHILDHOOD TRAUMA**			
	**Low**	**Medium**	**High**
Number of subjects (number of assessments)	229 (9,241)	258 (10,438)	201 (7,988)
% Females	71%	56%	53%
% Low education	2%	6%	8%
Mean age (*SD*)	17.8 (3.66)	17.4 (*SD* = 3.81)	17.6 (*SD* = 3.81)
Range	15–33	14–34	15–34
Mean trauma total score (*SD*)	27.2 (1.38)	32.4 (1.68)	43.1 (9.17)
**ASSESSMENT LEVEL**			
Cheerful mean (*SD* overall; between; within)	4.99 (1.49; 0.81; 1.26)	4.88 (1.47; 0.80; 1.24)	4.56 (1.63; 0.97; 1.32)^*^^†^
Insecure mean (*SD* overall; between; within)	1.64 (1.17; 0.60; 1.01)	1.70 (1.16; 0.65; 0.98)	1.82 (1.25; 0.72; 1.03)^*^
Relaxed mean (*SD* overall; between; within)	5.2 (1.48; 0.69; 1.31)	5.15 (1.43; 0.69; 1.26)	4.82 (1.53; 0.76; 1.34)^*^^†^
Anxious mean (*SD* overall; between; within)	1.4 (0.93; 0.45; 0.82)	1.46 (0.92; 0.48; 0.79)	1.60 (*SD* = 1.10; 0.63; 0.91)^*^^†^
Irritated mean (*SD* overall; between; within)	2.18 (1.62; 0.88; 1.36)	2.19 (1.52; 0.83; 1.29)	2.48 (1.66; 0.97; 1.36)^*^^†^
Down mean (*SD* overall; between; within)	1.59 (1.08; 0.55; 0.92)	1.69 (1.11; 0.64; 0.93)^*^	1.91 (1.27; 0.77; 1.02)^*^^†^
**GENETIC LIABILITY**			
	**Low liability**	**Intermediate liability**	**High liability**
Number of subjects (number of assessments)	452 (18,338)	90 (3,553)	56 (2,314)
% Females	59%	63%	71%
% Low education	6%	6%	10%
Mean age (*SD*)	17.5 (3.66)	16.4 (1.89)	17.9 (4.02)
Range	14–34	15–22	15–32
**ASSESSMENT LEVEL**			
Cheerful mean (*SD* overall; between)	4.85 (1.49; 0.84; 1.24)	4.76 (1.62; 0.91; 1.34)	4.56 (1.56; 0.93; 1.29)
Insecure mean (*SD* overall; between)	1.70 (1.16; 0.61; 0.99)	1.79 (1.29; 0.78; 1.06)	1.77 (1.24; 0.73; 1.01)
Relaxed mean (*SD* overall; between)	5.09 (1.45; 0.71; 1.28)	5.08 (1.47; 0.66; 1.32)	4.84 (1.54; 0.80; 1.32)^†^
Anxious mean (*SD* overall; between)	1.46 (0.93; 0.47; 0.81)	1.58 (1.11; 0.61; 0.95)	1.62 (1.12; 0.75; 0.86)
Irritated mean (*SD* overall; between)	2.26 (1.57; 0.89; 1.31)	2.40 (1.70; 0.87; 1.46)	2.29 (1.56; 1.03; 1.25)
Down mean (*SD* overall; between)	1.71 (1.12; 0.63; 0.94)	1.77 (1.21; 0.68; 1.01)	1.92 (1.35; 0.86; 1.05)

*SD, Standard deviation; DZ, Dizygotic twins; MZ, Monozygotic twins; Scl-90, Symptoms checklist*.

* *The difference in mean with that of the low subgroup is statistically significant*.

† *The difference in mean with that of the intermediate (or medium) subgroup is statistically significant*.

### Network graphs

**Figure 1:**
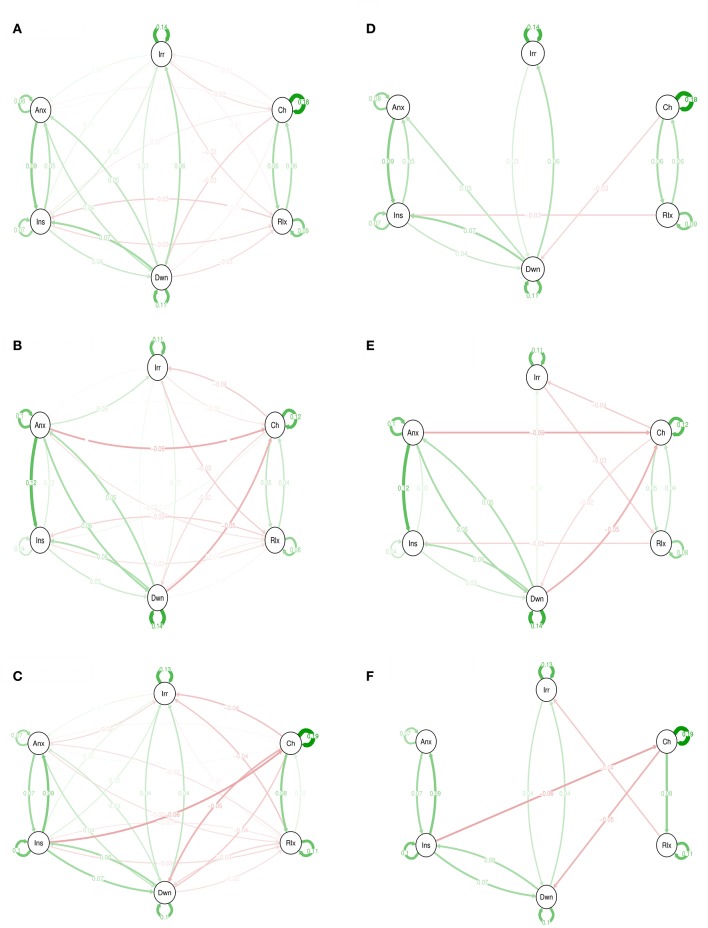
Emotions networks in subjects with low, medium, and high levels of childhood trauma. In this figure, the arrows represent associations over time; i.e., the B coefficient expressing the effect size of the predictive associations. For example, in the low CT network, there is an arrow from “relaxed” to “cheerful,” meaning that “relaxed” at *t*−1 predicts “cheerful” at t with a B coefficient of 0.06. Green arrows represent positive associations, and red arrows represent negative associations. The fading of the lines represents the strength of the association and are determined by the regression weights: the more solid the line, the stronger the association (and vice versa). Note that we can predict the emotion item from the previous state of the item itself. These arrows are the self-loops in the network. CT, childhood trauma. Graphs **(A–C)** are for low, medium, and high CT respectively. The Graphs **(D–F)** are for low, medium, and high CT respectively but only with associations that resisted to Simes correction for multiple testing with *p* < 0.022.

The networks in Figure 1 represent the associations between momentary emotion items for 3 levels of CT. For the sake of completeness, both the graphs with only edges that remained significant after Simes correction for multiple testing (i.e., with a *p* < 0.0224) and the complete networks are shown. For example, in the high trauma group, “insecure” at time point *t*−1 was negatively associated with “cheerful” at *t* (*B* = −0.06). Although, this was different from the regression coefficient in the other two trauma groups, significance disappeared after Simes correction. Thus, after Simes correction, none of the connections differed significantly between the strata.

**Figure 2:**
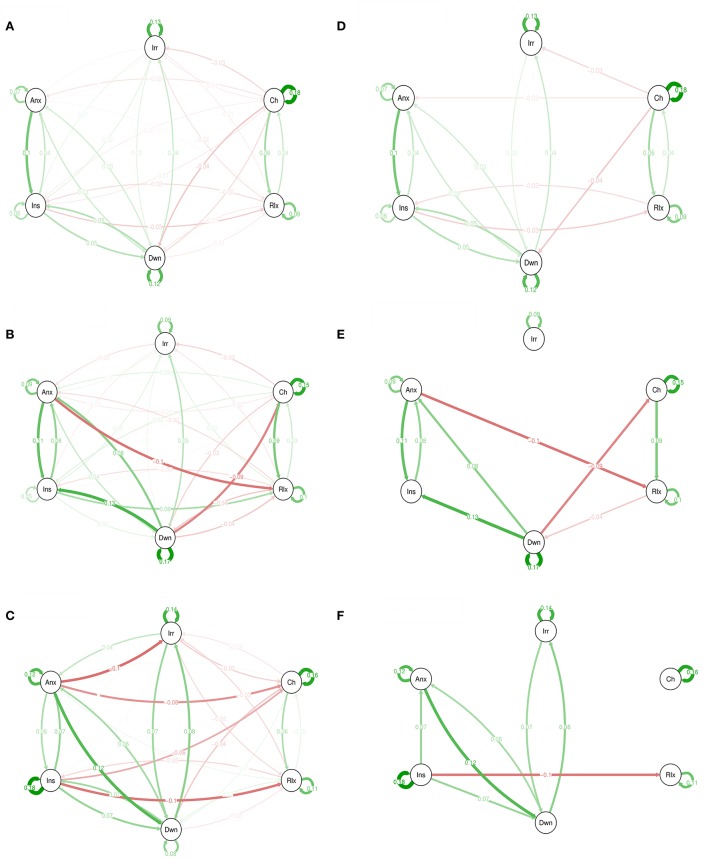
Emotions networks in participants with low **(A)**, intermediate **(B)**, and high genetic liability for psychopathology **(C)**. In this figure, the arrows represent associations over time; i.e., the B coefficient expressing the effect size of the predictive associations. For example, in the low genetic liability network, there is an arrow from “relaxed” to “cheerful,” meaning that “relaxed” at *t*−1 predicts “cheerful” at t with a B coefficient of 0.04. Green arrows represent positive associations, and red arrows represent negative associations. The fading of the lines represents the strength of the association and are determined by the regression weights: the more solid the line, the stronger the association (and vice versa). Note that we can predict the emotion item from the previous state of the item itself. These arrows are the self-loops in the network. Graphs **(A–C)** are for low, intermediate, and high GL respectively. The Graphs **(D–F)** are for low, intermediate, and high GL respectively but only with associations that resisted to Simes correction for multiple testing with *p* < 0.022.

Figure 2 shows network graphical representations for 3 levels of genetic liability. For example, in the high GL group, “insecure” at time point *t*−1 was associated with “insecure” at *t* (*B* = 0.18; self-loop).

### Structural characteristics of the networks

**Figure 3:**
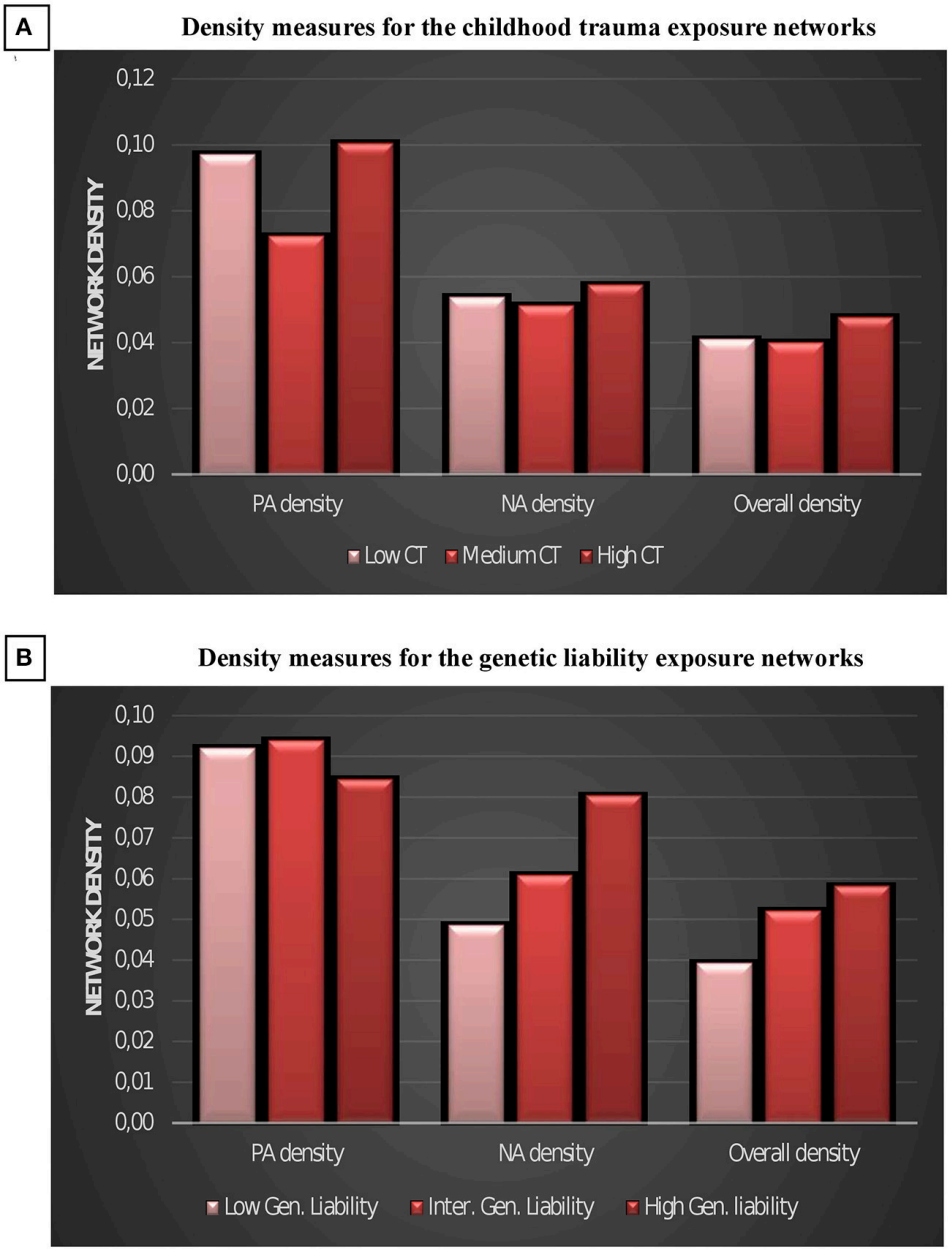
Density measures for the childhood trauma exposure emotions networks **(A)** and for the genetic liability emotions networks **(B)**. CT, childhood trauma; GL, Genetic liability; NA, negative affect; PA, positive affect.

PA density and overall density was higher in the high childhood trauma than in the intermediate trauma network (Table 2, Figure 3), but density did not linearly increase with increasing level of trauma. A linear increase was visible in negative and overall density between the GL strata, but only the difference between high and low GL was statistically significant (Table 2, Figure 3).

**Table 2:** Emotional density across levels of childhood trauma and genetic liability, respectively.

	**Density values**			***P*****-values of comparison from Permutation tests**		
	**Low CT**	**Medium CT**	**High CT**	**Medium vs. Low CT**	**High vs. Low CT**	**High vs. Medium CT**
PA density	0.09	0.07	0.1	0.06	0.86	0.02^*^
NA density	0.05	0.05	0.06	0.7	0.6	0.36
Overall density	0.04	0.04	0.05	0.82	0.08	0.04^*^
	**Low gen. liability**	**Inter. gen. liability**	**High gen. liability**	**Inter. vs. low GL**	**High vs. low GL**	**High vs. inter. GL**
PA density	0.09	0.09	0.08	0.89	0.66	0.66
NA density	0.05	0.06	0.08	0.22	0.00^*^	0.09
Overall density	0.04	0.05	0.06	0.05	0.02^*^	0.61

* *p < 0.05*.

“Cheerful” and “down” were most central with respect to outward strength, and cheerful was most central with respect to inward strength across all strata (Tables 3, 4). Centrality of the other emotional items differed between the strata of childhood trauma and the strata of GL, but there was no visible pattern, despite some statistically significant differences. Comparing edges separately, high GL participants showed a significantly stronger “insecure” self-loop than participants with low GL and participants with intermediate GL, but only the difference between high and intermediate GL survived Simes correction (Table 5). Only one other connection also survived Simes correction; in the high GL group, “insecure” was followed by a decrease in “relaxed” the next moment (the negative association of “insecure” at *t*−1 with “relaxed” at *t*; high vs. low; Table 5).

**Table 3:** Node strength centrality across levels of childhood trauma.

	**Centrality values**			***P*****-values of comparison from Permutation tests**		
	**Low CT**	**Medium CT**	**High CT**	**Medium vs. Low CT**	**High vs. Low CT**	**High vs. Medium CT**
**INWARD STRENGTH**						
Irritated	0.24	0.20	0.30	0.43	0.32	0.09
Cheerful	0.27	0.31	0.32	0.46	0.36	0.88
Relaxed	0.23	0.20	0.27	0.54	0.52	0.21
Down	0.25	0.29	0.33	0.43	0.11	0.40
Insecure	0.28	0.25	0.27	0.49	0.71	0.77
Anxious	0.20	0.20	0.23	0.97	0.54	0.49
**OUTWARD STRENGTH**						
Irritated	0.24	0.17	0.21	0.20	0.49	0.54
Cheerful	0.31	0.23	0.37	0.15	0.20	0.01^*^
Relaxed	0.20	0.17	0.24	0.51	0.44	0.18
Down	0.31	0.34	0.30	0.74	0.88	0.60
Insecure	0.21	0.14	0.37	0.22	0.02^*^	0.00^*^
Anxious	0.21	0.39	0.23	0.01^*^	0.89	0.01^*^

* *p < 0.05*.

**Table 4:** Node strength centrality indices and their relation to genetic liability to psychopathology.

	**Centrality values**			***P*****-values of comparison from Permutation tests**		
	**Low gen. liability**	**Inter. gen. liability**	**High gen. liability**	**Inter. vs. low GL**	**High vs. low GL**	**High vs. inter. GL**
**INWARD STRENGTH**						
Irritated	0.23	0.21	0.37	0.34	0.37	0.16
Cheerful	0.27	0.29	0.36	1.19	0.67	0.64
Relaxed	0.22	0.41	0.31	0.01^*^	0.59	0.16
Down	0.28	0.31	0.40	0.70	0.14	0.37
Insecure	0.25	0.34	0.34	0.24	0.44	0.89
Anxious	0.17	0.31	0.31	0.03^*^	0.11	1.07
**OUTWARD STRENGTH**						
Irritated	0.20	0.15	0.31	0.18	0.29	0.05
Cheerful	0.34	0.35	0.28	1.22	0.21	0.36
Relaxed	0.18	0.23	0.20	0.53	1.16	0.52
Down	0.28	0.56	0.32	0.00^*^	0.91	0.01^*^
Insecure	0.21	0.23	0.49	1.17	0.02^*^	0.02^*^
Anxious	0.21	0.36	0.50	0.25	0.05	0.40

* *P < 0.05*.

**Table 5:** Significant edge differences across different levels of GL.

**Differences**							**Coefficients**					
**Edges**	**Low vs. intermediate GL**		**Low vs. high GL**		**Intermediate vs. high GL**		**Low GL**		**Intermediate GL**		**High GL**	
	**Difference**	***p***	**Difference**	***p***	**Difference**	***p***	**B**	***p***	**B**	***p***	**B**	***p***
Insecure *t*-1 → Insecure *t*,	0.01	00.87	−0.12	00.00^*^	−0.13	00.01	00.06	00.00^†^	00.05	00.11	00.18	00.00^†^
Insecure *t*-1 → Relaxed *t*.	−0.09	00.01	00.06	00.14	0.16	00.00^*^	−0.03	00.01^†^	00.06	00.05	−0.10	00.01^†^

* *P < 0.0004*,

† *P < 0.02*.

*Simes corrected alpha for differences across subgroups is 0.0004 and for edge significance is 0.022*.

## Discussion

Using a dynamic network approach, we compared the time-lagged network structures across genetic and environmental risk strata. The primary goal of the study was to identify the impact of CT as an early environmental factor, and GL as a proxy genetic factor, on the structure of a time series network of six emotions—“irritated,” “cheerful,” “relaxed,” “down,” “insecure,” and “anxious”—at the levels of emotion density, node strength centrality and individual connections (edges). The principal findings were: (i) compared with the low GL stratum, the high GL stratum had significantly denser overall and negative emotion networks, while the medium GL stratum also showed a directionally similar but statistically insignificant association with network density; (ii) in contrast to GL, the results of the CT analysis were essentially inconsistent with our initial hypothesis; (iii) after adjusting for multiple testing, the individual edge comparisons across strata of GL and CT yielded only very few significant results.

### Genetic liability: the emotion network density

Considering the network density across different levels of GL, our current findings suggest an increase in overall and negative density as a function of the extent of GL. As far as we know, differences in density depending on GL have not been studied before. The current study partially replicates an earlier analysis (Hasmi et al., submitted), in which we observed a significant difference in overall density and negative density between high GL and medium GL without a linear increase in density values across the three strata, as opposed to the difference between high GL and low GL with a dose response relation in the present data (Supplementary Material). Also, the individual node comparisons across strata of GL yielded only no significant results (vs. two connections in the present data), after adjusting for multiple testing.

To the degree that higher network density may predict greater symptomatic severity under a high genetic loading, some studies are in apparent agreement with the present results. First, a denser cross-sectional network at baseline was associated with the persistence of clinical depression (van Borkulo et al., 2016; Wichers et al., 2016). Second, in analyses using ESM data, patients with depression, compared to healthy controls, had a higher overall density and negative density, but not a higher positive density (Pe et al., 2015). In agreement with this, higher levels of neuroticism have been associated with a denser emotion network (both overall and negative but not positive; Bringmann et al., 2016). Although, there was no direct estimation of density, several other studies also showed that the more a person shifts toward severe states of psychopathology, the stronger the regression coefficients of mental states at *t*−1 predict mental states at t (Höhn et al., 2013; Wigman et al., 2013). Moreover, according to the results of a recent study that investigated momentary assessed mental states and daily stress while generating three temporal networks in three groups of participants: patients with psychosis, their first-degree relatives, and healthy controls, the number of significant network connections increased in the group of patients with higher familiar risk for psychosis (Klippel et al., 2017). Which is relatively in accordance with the results of the present study; if we also consider the fact that, in that work, also connections with non-emotion-related items, e.g., being alone and being active were counted, and that the significance of the connections was not corrected for multiple testing as it is the case in the current work.

Considering the exploratory nature of the time-lagged network analysis of the ESM data and our previous findings, in which we found both higher overall density and higher negative density in the high GL stratum than it was in the medium GL stratum with no difference between low and high GL strata, we err on the side of caution when interpreting the current findings that might be suggestive of an increase in the connectivity of emotions with increasing levels of GL. There might be several explanations for the inconsistency between the previous and the current study. First, consistent with the assumption of the network theory of psychopathology and with previous work on affect regulation, the expected high between-subject variation might be contributing to reduction in reproducibility (Kuppens et al., 2012). Second, it is plausible to speculate that the differences between characteristics of the two samples may have contributed to inconsistency—the previous study consisted only of female participants with a mean age of 27.7 years. Gender and age differences in terms of symptom profile, vulnerability factors, and epidemiologic features in mental disorders are well identified (van de Water et al., 2016). To the best of our knowledge, there exists no network analysis of ESM data investigating the influence of age and only one study examining a gender effect in a sample of patients with major depressive disorder (MDD) and healthy controls, which showed that women with MDD had a denser negative emotion network than men with MDD, while the gender effect was not observed in healthy controls (Pe et al., 2015). In fact, these data—or lack thereof—indicate that there is a need to investigate the impact of basic demographic parameters (e.g., age and gender) on emotion networks before progressing to network analysis of mental disorder constructs in the context of vulnerability.

### Childhood trauma: the emotion network density

Regarding CT, findings were inconsistent, suggesting increased positive density and overall density in the high CT stratum compared to the medium CT stratum but not the low CT stratum, while negative emotion density did not differ across CT strata. In contrast to the current findings, our previous study showed that negative density in the high CT was significantly higher than the medium but not the low CT, with no significant differences in positive and overall emotion density measures across CT strata (see Supplementary Material).

### Structural characteristics of the networks

Similarly, the individual edge comparisons across strata of GL and CT yielded only very few significant and relatively inconsistent findings after adjusting for multiple testing with Simes correction to avoid spurious conclusions. In the previous female-female twin sample study, statistical comparisons between edges were also inconclusive. Regarding centrality comparisons, only the analysis of the “insecure” node across CT strata yielded a consistent pattern in terms of outward strength, replicating our earlier study. Feeling insecure -also studied as “uncertainty”- was found to be a powerful stressor in previous studies (Greco and Roger, 2003). In previous experiments with replicated results, informing the participants of a low probable electric shock induced more anxiety both at the emotional and physiological level (heart rate and skin conductance) than when the announced probability of the shock was 100% (Lewis, 1966; Epstein and Roupenian, 1970). The replicated finding of “insecure” differences across GL groups may support the notion of a genetic link between “insecure” and negative affect. It may be hypothesized that risk genes impact a brain circuit mediating negative emotion regulation and possibly more specifically emotional reactivity to feeling “insecure.”

### Strengths and limitations

The present study replicated the methodology of a recent paper (Hasmi et al., submitted) similar to a series of studies applying network analysis to intensive time series data obtained with ESM to gain insight into dynamic changes in mental states (Bringmann et al., 2013; Klippel et al., 2017). A large number of observations, inherent to the nature of ESM methodology, enabled us to compare three strata of both environmental and genetic exposures. Two other strengths are the use of permutation analyses as a correction for not including all random slopes and the subsequent correction of the alpha for multiple testing for both the p values of the significance of the regression coefficients and the comparison of those regression coefficients individually across networks. Such an approach proved useful, but it may have negatively affected statistical power and led to type-II error.

There were several limitations. First, only a limited set of momentary emotional mental states was included to overcome convergence problems in the analyses. However, the interest of studying affective mental states was served by this approach. An advantage of analyses in a limited set is also that network graphs are easier to interpret. Second, as data were initially collected from the general population, negative emotion items were relatively rarely reported by participants compared to a clinical population, and, thus, subject to floor effects. This limitation was dealt with by choosing items with the maximum moment-to-moment variation. Third, considering that our participants were young, mainly students, and living with their parents, the results of this study may not be representative of the overall population. Also, the network comparability could be biased by differences in means of emotion items and within-person variances. Means, however, are mostly analogous across GL strata and between the low and medium CT exposure. It hence seems improbable that differences in connection strengths and consequently in network indices, between these latter groups could be attributed to differences in variances. In contrast, the means in the group under high CT exposure are, for most of the emotion items, significantly different from the two other subgroups. Therefore, this could have been part of the reason explaining the lack of replicability between the two studies regarding network density under CT exposure.

Finally, this study is one of few that aimed to compare time series network despite the lack of specific and a valid methodology balancing both type I and type II errors. Further methodological studies are needed; these could, for example, test other advanced methods previously used in comparing cross-sectional networks, and replicate them in ESM based networks across different samples (Fried and Cramer, 2017). Additionally, future work may benefit from a dynamic process approach based on percolation theory (Shang, 2014) and multiplex network models, where interplay between and within several layers (e.g., emotions and vulnerability factors) can be more accurately modeled (Shang, 2015).

## Conclusion and future work

The present results represent a partial replication of previous work. The micro-level approach to what could be the phenotypic translation of the genetic liability to psychopathology was demonstrated in both samples, providing a potential link with negative emotion density. The fact that genes impact on the extent to which negative emotions impact each other is important as it helps to expose the complex ways by which genes are affecting mental health. These findings have relevance for future research in psychiatric genetics. First, it may help to explain current problems with replications across studies and, second, it may shine light on the need for novel designs that can take into account the complexity of genetic influence on the development of psychopathology.

## Author contributions

LH contributed to the conception of the work, the analysis, interpretation of data for the work, and to drafting it. MD contributed to the conception of the work, the analysis, interpretation of data for the work and to drafting and revising it. SG contributed to the interpretation of data for the work, drafting and revising it. CM, JD, RvW, DC, PD, MD, CD, ET, NJ, and BR contributed to the acquisition of data for the work, and to revising it. MW contributed to the conception of the work, the acquisition, the interpretation of data for the work and to revising it. JvO contributed to the conception of the work, the interpretation of data for the work and to revising it.

### Conflict of interest statement

The authors declare that the research was conducted in the absence of any commercial or financial relationships that could be construed as a potential conflict of interest.

## Abbreviations

CT: Childhood Trauma
GL: genetic liability
EFPTS: East Flanders Prospective Twin Study
ESM: Experience Sampling Method
NA: Negative Affect
PA: Positive Affect
SCL-90-R: Symptom Checklist-90-R
SD: Standard Deviation
MZ: Monozygotic
DZ: Dizygotic.
