# Supplementary material for: Network Approach to Understanding Emotion Dynamics in Relation to Childhood Trauma and Genetic Liability to Psychopathology: Replication of a Prospective Experience Sampling Analysis
Source: Front Psychol. 2017 Nov 2;8:1908. doi: 10.3389/fpsyg.2017.01908 (PMC5673657; doi:10.3389/fpsyg.2017.01908)
Supplement: Supplementary file 1 [file Table1.docx]

***Supplementary Material***

**Network Approach to Understanding Emotional Dynamics in Relation to Childhood Trauma and Genetic Liability to Psychopathology: Replication of a Prospective Experience Sampling Analysis**

**L. Hasmi*****, M. Drukker, S. Guloksuz, C. Menne-Lothmann, J Decoster, R. van Winkel, D. Collip, P. Delespaul, M. De Hert, C. Derom, E. Thiery, N. Jacobs, B. P. F. Rutten, M. Wichers, J. van Os**

*** Correspondence :** Laila Hasmi : l.hasmi@maastrichtuniversity.nl

**Preamble:** The results presented below are from supplementary analysis done upon the original network study twins sample [Hasmi, L., Drukker, M., Sinan, G., Viechtenbauer, W., Thiery, E., Derom, C., van Os, J. (2016). Genetic and environmental influences on the effective regulation network: A prospective experience sampling analysis. Manuscript submitted for publication].

**Table S1. Emotional density across levels of childhood trauma and genetic liability to psychopathology, respectively**

|  | Density values | | | P-values of comparison from Permutation tests | | |
| --- | --- | --- | --- | --- | --- | --- |
|  | Low CT | Medium CT | High CT |  | | |
|  |  |  |  | Medium vs Low CT | High vs Low CT | High vs Medium CT |
| PA density | 0.15 | 0.14 | 0.14 | 0.54 | 0.53 | 0.97 |
| NA density | 0.06 | 0.06 | 0.08 | 0.87 | 0.05 | 0.04* |
| Overall density | 0.05 | 0.05 | 0.06 | 0.69 | 0.12 | 0.06 |
|  | Low Gen. Liability | Inter. Gen. Liability | High Gen. liability | Inter. vs Low GL | High vs Low GL | High vs Inter. GL |
| PA density | 0.15 | 0.11 | 0.13 | 0.11 | 0.32 | 0.53 |
| NA density | 0.06 | 0.05 | 0.08 | 0.11 | 0.29 | 0.03* |
| Overall density | 0.06 | 0.05 | 0.06 | 0.03* | 0.87 | 0.03* |

* p<0.05

**Table S2. Node strength centrality across levels of childhood trauma**

|  | Centrality values | | | P-values of comparison from Permutation tests | | |
| --- | --- | --- | --- | --- | --- | --- |
|  | Low CT | Medium CT | High CT | Medium vs Low CT | High vs Low CT | High vs Medium CT |
| Inward strength |  |  |  |  |  |  |
| Irritated | 0.20 | 0.20 | 0.32 | 1,00 | 0.09 | 0.08 |
| Cheerful | 0.45 | 0.41 | 0.47 | 0.62 | 0.75 | 0.41 |
| Relaxed | 0.36 | 0.38 | 0.35 | 0.76 | 0.92 | 0.70 |
| Down | 0.34 | 0.34 | 0.45 | 0.99 | 0.05 | 0.06 |
| Insecure | 0.35 | 0.32 | 0.31 | 0.62 | 0.59 | 0.93 |
| Anxious | 0.23 | 0.22 | 0.27 | 0.87 | 0.55 | 0.46 |
| Outward strength |  |  |  |  |  |  |
| Irritated | 0.17 | 0.16 | 0.16 | 0.86 | 0.83 | 0.96 |
| Cheerful | 0.41 | 0.41 | 0.37 | 0.93 | 0.48 | 0.56 |
| Relaxed | 0.33 | 0.27 | 0.32 | 0.34 | 0.80 | 0.44 |
| Down | 0.49 | 0.48 | 0.67 | 0.86 | 0.06 | 0.06 |
| Insecure | 0.30 | 0.19 | 0.27 | 0.13 | 0.72 | 0.29 |
| Anxious | 0.23 | 0.36 | 0.40 | 0.20 | 0.09 | 0.75 |

**Table S3. Node strength centrality indices and their relation to genetic liability to psychopathology**

|  | Centrality values | | | P-values of comparison from Permutation tests | | |
| --- | --- | --- | --- | --- | --- | --- |
|  | Low Gen. Liability | Inter. Gen. Liability | High Gen. liability | Inter. vs Low GL | High vs Low GL | High vs Inter. GL |
| Inward strength |  |  |  |  |  |  |
| Irritated | 0.23 | 0.17 | 0.35 | 0.10 | 0.98 | 0.18 |
| Cheerful | 0.45 | 0.37 | 0.53 | 0.14 | 0.66 | 0.15 |
| Relaxed | 0.39 | 0.37 | 0.32 | 0.20 | 0.09 | 0.91 |
| Down | 0.35 | 0.38 | 0.49 | 1,29 | 0.15 | 0.18 |
| Insecure | 0.33 | 0.27 | 0.34 | 0.37 | 1,04 | 0.44 |
| Anxious | 0.25 | 0.30 | 0.28 | 0.77 | 0.82 | 0.89 |
| Outward strength |  |  |  |  |  |  |
| Irritated | 0.16 | 0.16 | 0.18 | 1,61 | 1,24 | 0.61 |
| Cheerful | 0.41 | 0.24 | 0.44 | 0.02* | 0.93 | 0.05 |
| Relaxed | 0.30 | 0.46 | 0.21 | 0.08 | 0.09 | 0.02* |
| Down | 0.59 | 0.42 | 0.66 | 0.17 | 0.50 | 0.10 |
| Insecure | 0.17 | 0.33 | 0.44 | 0.42 | 0.01* | 0.22 |
| Anxious | 0.36 | 0.23 | 0.31 | 0.05 | 0.32 | 0.46 |

*** p<0.05**
